# Association between sea lice (*Lepeophtheirus salmonis*) infestation on Atlantic salmon farms and wild Pacific salmon in Muchalat Inlet, Canada

**DOI:** 10.1038/s41598-018-22458-8

**Published:** 2018-03-05

**Authors:** Omid Nekouei, Raphael Vanderstichel, Krishna Thakur, Gabriel Arriagada, Thitiwan Patanasatienkul, Patrick Whittaker, Barry Milligan, Lance Stewardson, Crawford W. Revie

**Affiliations:** 1 University of Prince Edward Island, Atlantic Veterinary College, Department of Health Management, Charlottetown, C1A 4P3 Canada; 2 Interdisciplinary Center for Aquaculture Research (INCAR), University of Concepción, Concepción, 4030000 Chile; 3 Grieg Seafood BC Ltd., 1180 Ironwood Street, Campbell River, BC V9W 5P7 Canada; 4 Cermaq Canada Ltd., 919 Island Hwy #203, Campbell River, BC V9W 2C2 Canada; 5 Mainstream Biological Consulting, 1310 Marwalk Crescent, Campbell River, BC V9W 5X1 Canada

## Abstract

Growth in salmon aquaculture over the past two decades has raised concerns regarding the potential impacts of the industry on neighboring ecosystems and wild fish productivity. Despite limited evidence, sea lice have been identified as a major cause for the decline in some wild Pacific salmon populations on the west coast of Canada. We used sea lice count and management data from farmed and wild salmon, collected over 10 years (2007–2016) in the Muchalat Inlet region of Canada, to evaluate the association between sea lice recorded on salmon farms with the infestation levels on wild out-migrating Chum salmon. Our analyses indicated a significant positive association between the sea lice abundance on farms and the likelihood that wild fish would be infested. However, increased abundance of lice on farms was not significantly associated with the levels of infestation observed on the wild salmon. Our results suggest that Atlantic salmon farms may be an important source for the introduction of sea lice to wild Pacific salmon populations, but that the absence of a dose response relationship indicates that any estimate of farm impact requires more careful evaluation of causal inference than is typically seen in the extant scientific literature.

## Introduction

Increasing global demand for seafood and declining ocean fisheries have led to rapid growth in the aquaculture industry, including salmon farming, over the past few decades^1–4^. Farm-based production has been successful in meeting global demands and in generating revenue^1^; however, it has often been criticized for its potential negative impacts on the ecosystem and the interaction with valuable wild species through disease transmission, interbreeding, and competition^5–7^.

Pacific salmon (*Oncorhynchus* genera) are key species with strong cultural, socioeconomic, recreational, and symbolic significance to the residents of the Pacific Northwest^8,9^. In recent decades, the productivity of some Pacific salmon species has been decreasing^10–13^. Several factors are hypothesized to be associated with this decline, such as climate change, infectious diseases, anthropogenic impacts, or poor stock management, as well as the potential interplay among these factors^14^.

The spillover of various pathogens from non-native Atlantic salmon farms to sympatric wild fish has been a contentious issue since the onset of aquaculture in British Columbia (BC) in the 1970s. The transmission of sea lice between farmed and wild juvenile salmon along their migration routes has drawn particular attention, especially through social media, over the past years in BC^15,16^. Sea lice (*Lepeophtheirus salmonis* and *Caligus* species) are natural ectoparasites of salmonids, with a wide distribution in marine waters of the northern hemisphere^16,17^. Infestations can disrupt salmon normal behavior and growth, and cause mortalities in severe cases^17^. However, the effect that sea lice can have on out-migrating juvenile salmon is highly dependent on the size of the smolts^18,19^. In addition, it has been demonstrated that the *L. salmonis* found in the Pacific region differ from those in the North Atlantic and it has been hypothesised that, “nuclear and mitochondrial genetic changes that may help to explain apparent phenotypic differences observed between these forms”^20^. Sea lice are a major concern in salmon-producing countries, including Canada, with costs to the salmon industry amounting to millions of dollars annually^21^.

**Figure 1:**
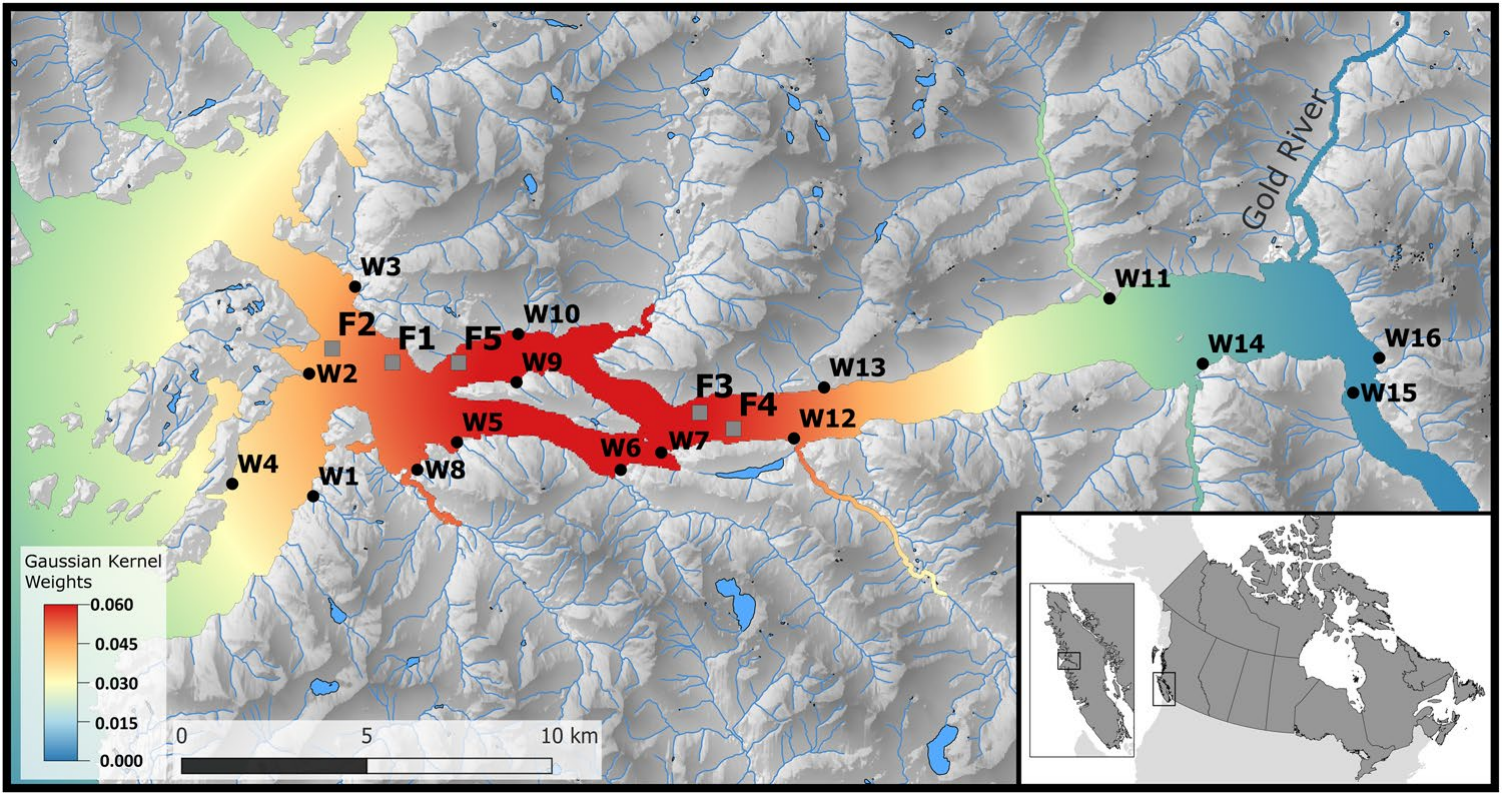
Locations of the study farms (grey squares; F1–F5) and the sampling sites for wild out-migrating salmon (black circles; W1–W16) within the Muchalat Inlet, BC, Canada, between 2007 and 2016. The west of the inlet opens to the Pacific Ocean. The water surface map is a visual representation of the Gaussian kernel weights, as determined from the five farm locations. The kernel densities were calculated by simulating a point process to represent a kernel density with a 30 km bandwidth. The points were simulated with ‘splancs’ package, and the kernel density surface with ‘spatstat’ package in R version 3.3.2 (http://www.R-project.org), and the maps were generated with QGIS version 2.18.13 (http://www.qgis.org).

A large group of researchers, environmental activists, and indigenous people believe that sea lice originating on Atlantic salmon farms are a key component in the putative decline in some Pacific salmon stocks in BC^22–25^. A number of studies, focused on the Broughton Archipelago region, present contradictory evidence for the impact of sea lice infestation at the interface between farmed and wild salmon in BC. For instance, Marty *et al*. could not find any associations between sea lice abundance on farms and the productivity of wild salmon populations in Broughton Archipelago^26^; whereas other researchers have demonstrated a negative correlation^23,25^. In addition, sea lice abundance in both farmed and wild populations shows prominent temporal and geographic variability^27,28^. Hence, in order to adopt the most efficient regional strategies for controlling sea lice, it is necessary to explain the spatiotemporal patterns of the infestation for each specific system, with respect to its unique distribution of wild species, microclimate, and oceanographic characteristics. In the present research, we focus on the Muchalat Inlet, BC, located on the west coast of Vancouver Island (Fig. 1). The first salmon aquaculture farm in the region was stocked in late 2003. Since then, sampling of farmed and wild juvenile salmon for the monitoring of sea lice levels has been in place. *L. salmonis* is the dominant species of sea lice in this region^29^ and the focus of the current study. The geographical isolation, access to the sea lice infestation data on both farmed and wild salmon, and the dominance of one species of wild Pacific salmon, i.e. Chum (*O. Keta*), in Muchalat Inlet provided an ideal setting for conducting our study of the host-parasite dynamics within this aquatic ecosystem. Two studies have been conducted previously by our group in collaboration with partners from the Muchalat Inlet on sea lice infestation and its associated determinants on farmed salmon^30^ and wild juvenile salmon^29^, but neither of those studies looked at the potential transmission of sea lice between farmed and wild salmon. In addition, our access to high quality data over the last few years is another advantage that motivated us to conduct the current study. The objective of this study was to evaluate the potential association between sea lice (*L. salmonis*) infestation observed on Atlantic salmon farms and those on sympatric wild out-migrating Pacific salmon in the Muchalat Inlet region of BC.

## Results

### Descriptive statistics

**Figure 2:**
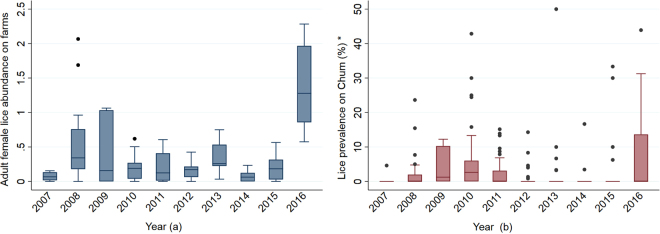
(**a**) box-plot for the mean abundances of adult female *L. salmonis* on farms, in Muchalat Inlet, limited to the February-May (t-1) window for each study year (2007–2016; n = 140); (**b**) box-plot for the distribution of the prevalence of infestation with *L. salmonis* on out-migrating Chum (%) in Muchalat Inlet, during the March–June (t) window for each study year. Prevalence values were estimated for each wild site-year-month instance (n = 365). Each box represents the interquartile range including the median line. Whiskers represent the lowest and highest adjacent values. Small circles represent outliers. *In plot (**b**), two outliers for lice prevalence have been removed to improve clarity in visualising the distributions. These two values of 100% and 75% occurred with very low sample sizes (3/3 in 2010, and 3/4 in 2016, respectively).

For the farm data, 410 analytical units were available. The annual abundance of adult female *L. salmonis* and the number of salmon sampled at the study farms are presented in Table 1. Overall, 27,163 fish were evaluated during the study period from all farms, to which a total of 12,947 adult female *L. salmonis* were attached. The highest and the lowest numbers of fish were sampled in 2011 (n = 3968) and 2016 (n = 1263), respectively. The median abundance of adult female lice on salmon farms in the Muchalat Inlet, during the February-May window, was considerably higher in 2016 (1.30) than in previous years, which did not show marked variability in median abundance, with values ranging between 0.05 and 0.35 (Fig. 2a).

**Table 1:** Abundance of adult female *L. salmonis* and the number of sampled salmon (in parentheses) by study farm (F1–F5) across each year, between 2007 and 2016. A dash represents a fallow year (no sampling events) for a given farm.

Farm	2007	2008	2009	2010	2011	2012	2013	2014	2015	2016
F1	0.79 (729)	0.33 (678)	0.72 (568)	0.45 (729)	0.19 (1387)	0.25 (640)	—	—	1.28 (1001)	1.52 (362)
F2	0.26 (374)	0.71 (663)	0.57 (710)	0.87 (504)	0.12 (989)	0.17 (645)	0.42 (563)	0.17 (560)	0.71 (1041)	1.34 (301)
F3	—	0.16 (432)	0.43 (734)	0.20 (755)	0.23 (840)	—	—	0.18 (1042)	0.61 (724)	—
F4	0.14 (803)	0.48 (63)	—	—	—	0.27 (790)	0.43 (881)	0.54 (685)	0.60 (703)	—
F5	0.17 (589)	0.64 (783)	0.52 (368)	0.56 (1128)	0.30 (752)	0.37 (847)	0.11 (80)	0.99 (660)	1.18 (460)	0.83 (600)
All farms	0.15 (2495)	0.48 (2619)	0.55 (2380)	0.50 (3116)	0.20 (3968)	0.27 (2922)	0.41 (1524)	0.44 (2947)	0.87 (3929)	1.15 (1263)

The Chum salmon data included 365 analytical units, which formed the basis for our master dataset. After removing 85 mismatched values (randomly missing X_i,t_) from the farm data, the final master data set included 280 analytical units for the modeling procedures. The number of Chum sampled and infested (having at least one louse), during the study period, by sampling site, are presented in Table 2. As can be seen in this table, the overall trend in the number of collected fish decreased over the period, and the annual proportion of chum with any *L. salmonis* infestation varied between 1.7 and 4.6%, with the exception of 2016 where the proportion was 11.4%. The distribution of prevalence values, for each month at each sampling site, by study year is presented in Fig. 2b. In general, the median levels of sea lice prevalence on out-migrating Chum were very low between 2007 and 2016 (<4%); however, there was greater variability in the distribution seen in 2016, compared to previous years. This finding was consistent with higher abundance of sea lice on the farms in that year (Fig. 2).

**Table 2:** Distribution of the number of fish with *L. salmonis* counted on out-migrating Chum salmon by sampling site (infested/sampled Chum), between 2007 and 2016. A dash represents no sampling events. The overall annual proportion of infestation (%) across all sites is also given for each year (the last row).

Wild site	2007	2008	2009	2010	2011	2012	2013	2014	2015	2016
W1	1/328	4/308	0/342	13/253	2/357	0/109	3/64	1/38	9/32	1/17
W2	0/173	2/100	3/284	0/45	6/66	3/106	3/36	0/73	0/30	3/14
W3	2/76	3/130	16/265	4/225	3/252	1/196	0/60	0/70	1/65	7/82
W4	10/363	3/265	11/242	4/193	3/266	—	—	—	—	—
W5	0/148	5/223	8/274	7/188	5/178	5/63	0/90	0/38	0/4	18/42
W6	10/281	30/211	14/98	25/282	19/238	0/63	0/30	0/36	0/33	6/68
W7	18/183	0/4	—	—	—	0/8	0/1	0/86	0/4	7/32
W8	6/131	31/228	8/223	13/220	1/271	—	—	—	—	—
W9	0/91	1/55	—	—	—	5/257	2/91	0/43	3/60	12/59
W10	2/197	0/69	3/74	16/245	0/41	1/334	1/90	0/79	0/6	4/76
W11	0/155	15/87	5/127	1/97	0/70	1/174	0/90	1/114	0/95	2/90
W12	2/92	5/112	17/154	3/106	9/71	6/143	0/90	0/50	1/18	3/25
W13	0/56	0/63	13/195	1/31	6/164	0/114	0/60	0/78	0/43	0/46
W14	0/350	0/181	—	—	—	—	—	—	—	—
W15	0/113	0/72	—	—	—	—	—	—	—	—
W16	0/146	0/220	—	—	—	—	—	—	—	—
All sites (%)	1.7	4.2	4.3	4.6	2.7	1.4	1.2	0.3	3.6	11.4

### Analytical statistics (models)

**Figure 3:**
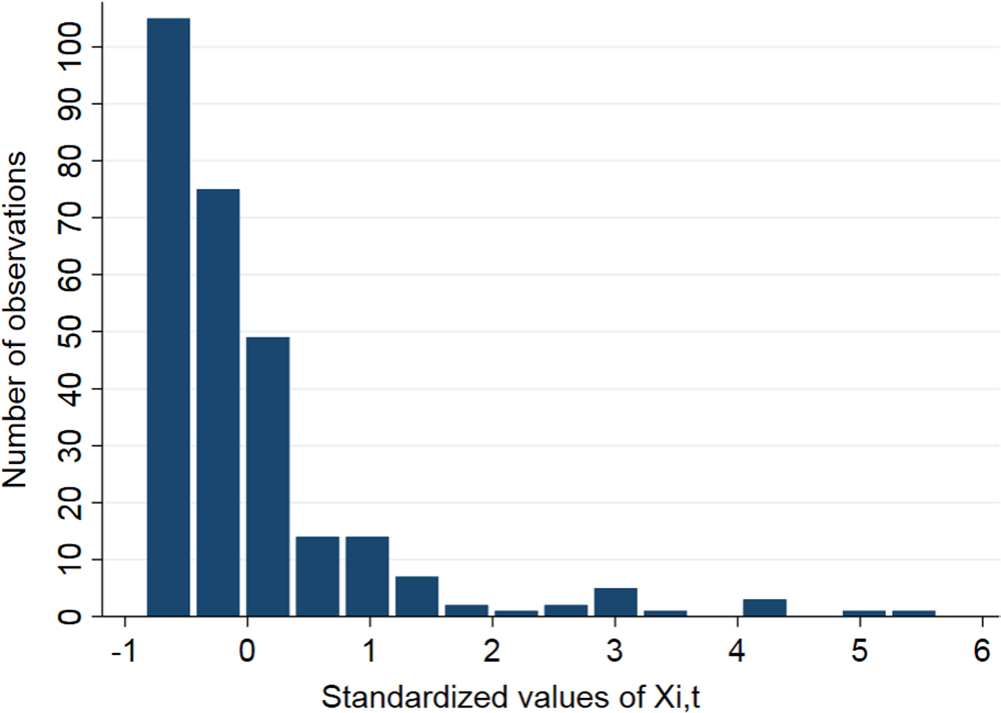
Frequency distribution of the overall standardised adult female *L. salmonis* output pressure from the study farms (X_i,t_; the main predictor of interest) in Muchalat Inlet, BC, from 2007 to 2016 (n = 280).

**Figure 4:**
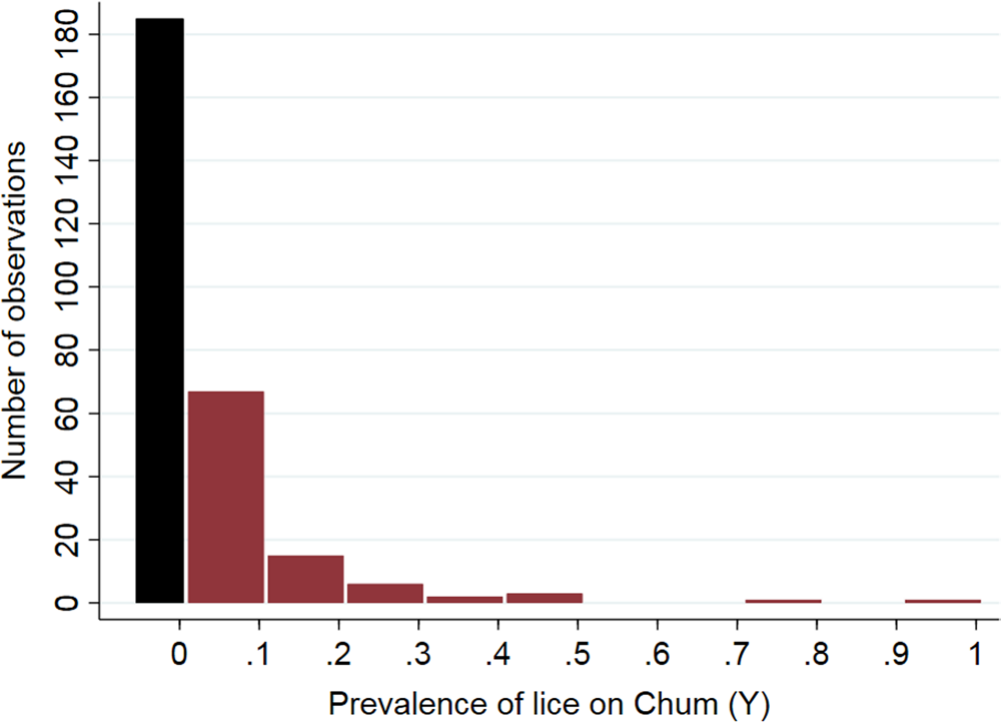
Frequency distribution of the prevalence of infestation with *L. salmonis* on out-migrating Chum salmon in Muchalat Inlet, BC, from 2007 to 2016. Each observation is a unique combination of wild site-year-month (n = 280). The black bar represents the number of zero prevalences (n = 185).

The main predictor of interest, X_i,t_, had a very wide range of values (28–126,613). It was, therefore, standardized (centered to its mean of 16,572, and divided by its SD of 19,507) to provide more meaningful interpretations (Fig. 3). The effect of wild sampling site on the outcome (Y; Fig. 4) was not statistically significant at any stage of the modeling process (P > 0.05); therefore, it was removed from the final models.

### Mixed-effects logistic model (Model 1)

This model was built upon the final data set (n = 280) to evaluate the association between the overall output pressure of lice from the farms (X_i,t_) and the log-odds of the presence of an infestation (Y). To meet the linearity assumption in the final model, a quadratic term for the main predictor of interest (X_i,t_^2^) was added to the model. Here is the final model equation:

$${\rm{Logit}}\,({\rm{P}})={\beta }_{0}+{\beta }_{1}({{\rm{X}}}_{{\rm{i}},{\rm{t}}})+{\beta }_{2}{({{\rm{X}}}_{{\rm{i}},{\rm{t}}})}^{2}+{\beta }_{3}({\rm{April}})+{\beta }_{4}({\rm{May}})+{\beta }_{5}({\rm{June}})+u$$

where, ‘P’ is the probability of infestation, with any lice, at any given ‘site-year-month’ (or the probability that wild-prevalence is non-zero); β_0_ is the constant; β_s_ are regression coefficients (Table 3); and ‘*u*’ is the random effect of ‘year’.

**Table 3:** Results for the final mixed-effects logistic regression model evaluating the effect of *L. salmonis* output pressure (X_i,t_) from the study farms on the log-odds of the presence of infestation with lice on out-migrating Chum salmon (Y) in Muchalat Inlet, during 2007–2016.

Variable	Coefficient	95% CI	P-value
Fixed effects			
X_i,t_	1.08	0.46; 1.70	0.001
X_i,t_^2 a^	−0.18	−0.35; −0.02	0.029
Month			<0.001^b^
March	Ref.^c^	—	—
April	2.27	1.29; 3.25	<0.001
May	2.32	1.29; 3.34	<0.001
June	0.38	−0.95; 1.71	0.576
Constant	−2.44	−3.49; (−1.39)	<0.001
Random effect variances	ICC^d^	95% CI	P-value
Year	0.21	0.07; 0.47	<0.001

^a^X_i,t_^2^ is the quadratic term for the main predictor of interest (X_i,t_: standardized output pressure of sea lice). The overall P-value for X_i,t_ and X_i,t_^2^ was 0.0019.

^b^The overall P-value for the month effect.

^c^Reference category.

^d^ICC: intra class correlation coefficient.

**Figure 5:**
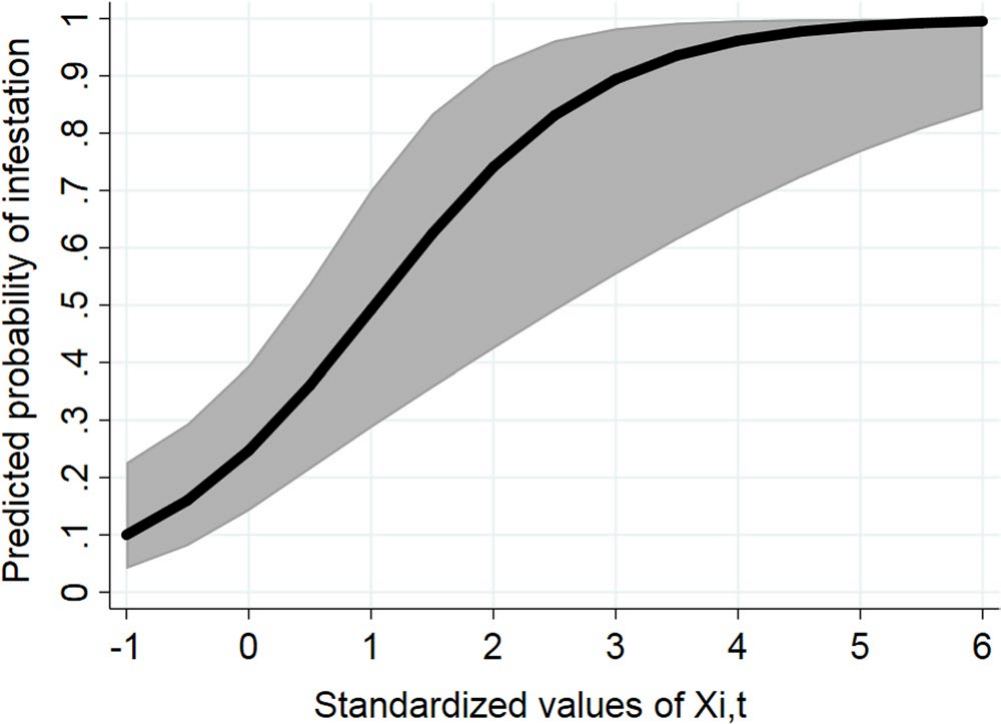
Margins plot (based on Model 1) illustrating the relationship between the standardized *L. salmonis* output pressure (the main predictor of interest, X_i,t_) from the study farms (X-axis) on the predicted probability of the presence of infestation with lice on out-migrating Chum salmon (Y-axis) in Muchalat Inlet between 2007 and 2016. The grey area represents 95% confidence interval about the prediction line (black).

Results for the mixed-effects logistic model are summarized in Table 3. Based on the model estimates, the relationship between changes in X_i,t_ and the predicted probability of the presence of any lice on out-migrating Chum is illustrated in Fig. 5. In general, as the overall output pressure of *L. salmonis* from farms (X_i,t_) in Muchalat Inlet increased, the probability for an infestation to occur on out-migrating Chum (Y) increased (Fig. 5). This increasing trend slows after nearly 3 standardized units of X_i,t_, which may indicate a point of saturation. The month effect was also a significant predictor for the presence of sea lice on Chum salmon (Table 3; P < 0.001). In April and May, there was a significant increase in the probability of infestation with *L. salmonis* on out-migrating Chum (Y), compared with March and June (Table 3).

Of the total unexplained variation in Y, 21% was attributed to the variation among the study years. As can be seen in Fig. 2(b), two high periods for the prevalence occurred between 2009 and 2010, and in 2016. These periods contributed more to the overall variability than other years with lower prevalences. The interaction between X_i,t_ and month was not significant (P = 0.42); therfore, it was not included in the final model.

### Mixed-effects linear model (Model 2)

This model was built using only the non-zero prevalence wild sampling events (n = 95) to evaluate the association between the overall output pressure of lice from the farms (X_i,t_) and the prevalence of infestation with lice (Y). To meet the assumption of normality, a logarithmic transformation was implemented on Y. Here is the final model equation:

$$\mathrm{Ln}\,({\rm{Y}})={\beta }_{0}+{\beta }_{1}({{\rm{X}}}_{{\rm{i}},{\rm{t}}})+{\beta }_{2}({\rm{April}})+{\beta }_{3}({\rm{May}})+{\beta }_{4}({\rm{June}})+u+\varepsilon $$

where, ‘Y’ is the prevalence of infestation at any given ‘site-year-month’ if non-zero; β_0_ is the constant; β_s_ are regression coefficients (Table 4); ‘*u*’ is the random effect of ‘year’; and ‘ɛ’ is the error term.

**Table 4:** Results for the final mixed-effects linear model evaluating the effect of *L. salmonis* output pressure (X_i,t_) from the study farms on the log-prevalence of the infestation on out-migrating Chum salmon (Y) in Muchalat Inlet, during 2007–2016.

Variable	Coefficient	95% CI	P-value
Fixed effects			
X_i,t_	0.11	−0.11; 0.33	0.326
Month			<0.001^a^
March	Ref.^b^	—	—
April	0.36	−0.31; 1.03	0.228
May	0.78	0.08; 1.48	0.029
June	2.01	0.98; 3.03	<0.001
Constant	−3.43	−4.14; −2.73	<0.001
Random effect variances	ICC^c^	95% CI	P-value
Year	0.21	0.05; 0.58	0.020

^a^The overall P-value for the month effect.

^b^Reference category.

^c^ICC: intra class correlation coefficient.

Results for the mixed-effects linear model have been summarized in Table 4. As shown in the table, association between the overall output pressure of *L. salmonis* from the farms (X_i,t_) and the prevalence of infestation with *L. salmonis* on out-migrating Chum (Y) was not statistically significant (P = 0.33). A significant positive association (P < 0.001) was found between the prevalence of *L. salmonis* on out-migrating Chum and the progress of the out-migration season (from March through June; Table 4). Similar to Model 1, approximately 21% of the total unexplained variation in Y was due to the years effect (Table 4). The interaction between X_i,t_ and month was not significant (P = 0.37).

## Discussion

This is the first time that the effects of farm-origin *L. salmonis* on wild salmon on the west coast of Vancouver Island, BC, have been studied. The geographical isolation of our study ecosystem and the relative dominance of one wild species (i.e. Chum) provided ideal conditions to investigate this relationship. Elmoslemany *et al*. investigated a number of site-specific determinants (e.g. salinity and temperature at sampling) of the infestation with sea lice on wild salmon at the fish level in Muchalat Inlet, during the period 2004 to 2011. However, that study did not include farms’ effects in the analyses. It has been proposed that *L. salmonis* is a density-dependent pathogen and that the density of salmonid farms surronding a site can have substantial impact on the sea lice abundance on a given farm^31,32^. Therefore, we conducted this population-level study that incorporated the potential lice spread from all of the farms in the study region, and defined an overall output pressure from the farms (i.e. the main predictor of interest), which could presumably drive infestation levels on sympatric out-migrating salmon. To achieve this, we applied a similar methodology to one previously described^33,34^, which defines the overall output pressure based on a combination of three influential factors: the farms’ operational sizes, *L. salmonis* abundance on each farm, and distances from the sampled wild sites.

Based on our logistic model, an increased load of adult female sea lice on farms was associated with a higher probability that infestation would be present on juvenile Chum in the region, indicating the important role that salmon farms can have in the introduction of sea lice to out-migrating wild fish. This finding is in agreement with other studies that have reported that salmon farms can act as a major source for sea lice to sympatric wild salmon along their migration paths^16,22–25,35^. However, based on our linear model, when infestation was present on wild fish, the extent of this infestation (i.e. the prevalence levels) was not associated with the farms’ output pressure. This latter finding may be a statistical artifact related to the limited number of observations that were available in the second model (n = 95), or the presence of two large outliers in the prevalence (as noted in the footnote of Fig. 2b). However, it may also be that biological factors such as an immune response in the Chum or active movement into low salinity environments help to control the level of infestation^36^. As such, prospective data collection and analyses may help refine the nature of the relationship between farm and wild infestation levels.

In general, the infestation levels with *L. salmonis* on the study farms were consistently at low levels and did not exceed the threshold regulated by the Fisheries and Oceans Canada (DFO) (i.e., three motile *L. salmonis* per fish) over the study period, which indicates the effectiveness of the farm-level control measures in the study region. In BC, during the out-migration season of wild juvenile salmon (March–June), if the regulatory threshold is exceeded, farmers must take appropriate management measures (i.e. harvesting or treatment) in order to reduce the risk of exposure at the interface of farmed and wild fish (www.dfo-mpo.gc.ca/aquaculture/protect-protege/parasites-eng.html). For instance, antiparasitic treatments (in the case of study farms in-feed, emamectin benzoate was used) were typically carried out during winter (prior to wild salmon out-migration) and/or summer (prior to the return of wild adults) (data not shown), in line with perceived best practice and as recommended in other research^25,37^.

The prevalence of infestation with sea lice on out-migrating Chum was also consistently at very low levels, though a significant rise was observed in 2016. This rise corresponded to a marked increase in the lice abundance on the study farms over the same time period. One main reason for the increased levels of infestation in 2016 could be the timing of lice treatments in 2015 and 2016. Three of the study farms were active from September 2015 through 2016 (Table 1), but lice treatment was implemented on only one farm (F5), in April 2016. This treatment was in the middle of the wild out-migration window; hence, perhaps it was not very efficient in reducing the output pressure from that farm. Moreover, the last lice treatments for the other two farms (F1 and F2) were carried out in August 2015, without any treatments in 2016. Therefore, the observed increase in the farms’ lice abundance between February and May, could have led to the increased lice prevalence on juvenile Chum in 2016. Another possible reason that could have contributed to the 2016 rise in the farm abundance of *L. salmonis* was the abnormally high temperatures during the winter of in BC^38^. Bateman *et al*. studied the abundance of *L. salmonis* on farmed and wild salmon in the Broughton Archipelago region, on the east coast of Vancouver Island, during 2001–2015. They detected outbreaks of this parasite on out-migrating Pink and Chum stocks in 2015 and concluded that the observed outbreaks were also associated with the improper timing of treatments and warmer than usual environmental conditions^22^. Furthermore, they suggested that the unusually high return of Pink salmon to the Broughton Archipelago region could have been another influential factor in 2015. We, however, did not have access to the wild salmon return data in the study region. In the Broughton Archipelago region of BC, the overall annual proportions of infestation with *L. salmonis* on juvenile Chum typically ranged between 10% and 17% over a similar period^39^, once again illustrating the relatively low levels of infestation typically seen in the Muchalat Inlet.

The effects of salinity on the development of *L. salmonis* and local infestation levels in regions of BC for this parasite have been established^30,40^. In line with our study objective, to account for the potential confounding effects of salinity and temperature on the relationship between the overall load of lice from the farms and the prevalence of infestation on wild fish, we included the month fixed-effects as well as the year random effects in our final models. Thus, the monthly variabilities in temperature and salinity at each wild sampling site were absorbed into the month’s effects (a surrogate role). Based on the first model, the probability of infestation of wild fish increased from March to May, but dropped in June. It has previously been shown that with increasing temperature towards summer months, along with the beginning of the in-migration of adult wild salmonids later in the summer, sea lice infestation levels can rise on both farms and wild fish^29,41^. Our contradictory finding of a lower probability of infestation in June compared to the previous months could be attributable to the limited number of samples in this month, which comprised only 13.4% of the total samples in the final data set. While Chum were the dominant species early in the out-migration period, Chinook (*O. tshawytscha*) and Coho (*O. kisutch*) were more likely to be observed in Muchalat Inlet^29^ later in the season. Another explanation for the lower levels of infestation in June could be the expected decline in salinity from June through November, in most years around Vancouver Island, due to the influence of freshwater; i.e. melting snow^29,42^.

Previous studies^33,34^ considered a maximum travel distance of 30 km for sea lice particles in terms of assessing their effect on neighboring farms or wild fish. We examined a range of biologically plausible distances and did not find any substantive differences in the fit of the models that used a range of bandwidths, from 30 to 60 km. Therefore, we chose the widely adopted distance of 30 km in our final analyses^42,43^. In common with those studies which used the overall output pressure, one of the challenges that existed for our study was that we were not able to account for the physical oceanographic features, such as tidal movements, due to the lack of information. In addition, other factors, such as biological behaviour of the larvae, wind, and short-term fluctuations in weather and temperature/salinity profiles, may substantially affect the oceanographic features and lice dispersal patterns and survival^15,42,44^. An additional limitation in our study was the use of Gaussian kernel density weights for seaway distances between the wild sampling sites and farms (d_i,j_). In this regard, it was assumed that the weight for any given distance around a site was equal (i.e. symmetrical radial weights), which may not adequately account for the duration of exposure of wild fish to sea lice particles at a site along their migration path. However, with respect to the fact that juvenile Chum may join at different points (river openings) along the Muchalat Inlet, the actual exposure time for any given group of fish at each sampling site was not known. By and large, Gaussian kernel weights have been deemed to provide reasonable approximations when applied in similar studies^33,34^.

In general, Pink and Chum are proportionally the most abundant wild salmon species around Vancouver Island^15,39^. We restricted our final analyses to Chum due to the very low proportion of other Pacific salmon species in Muchalat Inlet. On the one hand, this may limit the generalizability of our results to other wild salmon species; but on the other, it may increase the precision of our results (i.e. reduce sources of potential bias). In this regard, the known confounders associated with the species of fish, such as different biological behaviors, susceptibility to *L. salmonis*^45^, and migration size^29^, did not affect our results. Chum begin their out-migration in early March, once they emerged from the gravel. They quickly out-migrate through the river systems draining into Muchalat Inlet, and will usually reach the ocean within a few days^8,29^. Therefore, some of potential risk factors for infestation with *L. salmonis* during out-migration from fresh water to marine environment (e.g. smolt length) are unlikely to affect our analyses or cause substantial bias.

In this study, we focused on the role of farms as the main source of infestation for out-migrating juveniles. We should not ignore the prominent role that returning wild adults have in spreading sea lice to farms and other young wild Pacific salmon in the fall. We were not able to assess this association due to the lack of appropriate data on wild returns to Muchalat Inlet. This potential source of sea lice was not expected to directly affect the prevalence of infestation on out-migrating juveniles, as there is allopatric separation between the out-migrating Chum and the returning adults^8,46^. However, *L. salmonis* can be transferred from returning wild adults to other Pacific salmon species such as Coho, which may spend up to a year in the estuaries, and the resident Pacific salmon and trout, such as cutthroat and steelhead, in the vicinity of out-migration routes of juvenile Chum^8^. Various non-salmonid species, including herring and sticklebacks that overwinter in the coastal area, can also carry *L. salmonis*, though the significance of these sources is believed to be minor^33,47,48^.

Although several studies have pointed a finger at the growing salmon aquaculture industry over the past two decades as a major cause of putative decreases in the productivity of some wild salmon populations^4,16,22–25,35^, there remains controversy around the evidence involving interactions between farmed and wild salmon. Global climate change, anthropological manipulation of the environment, and emerging diseases can all play a role in any such declines. Studies to further elaborate the relative impacts of such factors are definitely needed.

## Conclusions

Our study found that population-level abundances of sea lice on farmed and wild salmon in Muchalat Inlet were very low. Our analyses suggest that farm-origin sea lice can influence the likelihood of *L. salmonis* being introduced to sympatric juvenile Chum. However, the levels of sea lice infestation observed on these wild fish did not appear to be influenced by the sea lice abundances recorded on farms. Therefore, continued compliance with the current regulations regarding sea lice control on the farms in BC should be an efficient strategy to avoid outbreaks of this parasite on the valuable wild stocks along their migration routes.

This study has shed some light on the controversy that exists around ecological impacts at the interface of farmed and sympatric wild salmon populations. However, additional observational studies in other BC farming areas over long periods of time (and preferably prospectively followed) are recommended, which could be based upon the general framework presented in the current study. Increased clarity around the nature of these ecological interactions is required to guide the sustainable growth of the salmon industry, while ensuring the successful preservation of valuable wild stocks.

## Methods

### Study area

This study was conducted in Muchalat Inlet, located on the west coast of Vancouver Island, BC (Fig. 1). There are five Atlantic salmon (*Salmo salar*) farms in this region, all belonging to one company. Farms in Muchalat Inlet are relatively isolated from other farms on Vancouver Island (the next nearest farm is located in channels to the northwest, more than 30 km by seaway). The sampling of wild out-migrating salmon for the monitoring of sea lice began in 2004 following the onset of salmon farming in Muchalat Inlet. A total of 16 sites were identified along the inlet and deemed suitable for sampling, based on distances from farms and the need for geographical representation^29^. Figure 1 indicates the study area, locations of the farms, and the sampling sites along the inlet.

### Data collection and management

The final data set for this research was obtained from three sources, as follows:

1. Farm data: provided by the farming company in Muchalat Inlet, from 2004 to 2016. The data set included sea lice counts, environmental (temperature and salinity), and production (weight and number of fish) variables. Sea lice data were recorded on a monthly basis at the cage level and consisted of *L. salmonis* counts at different life stages: pre-mobile, mobile (pre-adults and adult males), and adult females. In each month, 1 to 11 (mean: 4) cages and 19–101 fish (mean: 21) per farm were sampled. The farms were active at various time periods (production cycles) from 2004 to 2016. Because the farming activity was limited to only one farm between 2004 and 2007, we initiated our study with the 2007 data. More details on sampling and lice counting procedures have been presented elsewhere^30^. For our statistical analyses, the data were aggregated at the farm level and each analytical unit was defined as a unique combination of farm-year-month.
2. Wild salmon data: extracted from a database prepared by the Mainstream Biological Consulting and the Atlantic Veterinary College, University of Prince Edward Island. This data set included weekly, bi-weekly, or monthly sea lice counts on wild out-migrating (juvenile) salmon sampled from 16 designated sites along Muchalat Inlet using beach seines, during the study period (2007–2016). Sampling was carried out during the Pacific salmon out-migration season; i.e. from March through June (4 months per year) at various sites and points in time (Table 2). Since 2013, sampling has been limited to 3 months (March, April, and May) because of the low number of wild juveniles in June. Sampling at Sites 14, 15, and 16 was discontinued after 2008 due to consistent zero lice infestation levels in the previous years (2004 to 2008). Sampling protocols were developed in consultation with experienced researchers from DFO. More details on the sampling and laboratory procedures have been published elsewhere^29^. Because the majority (84%) of the wild fish sampled in the region between 2007 and 2016 were Chum (*Oncorhynchus keta*), we further limited our data analyses to this species. Due to scarcity of the weekly data, and to be consistent with the farm-level data, the merging process aggregated the data at the month level; therefore, each analytical unit represented a unique combination of site-year-month.
3. Seaway distances: seaway distances between each combination of wild sampling sites and farms were calculated with the ‘gdistance’ package^49^ in the R statistical language^50^, using each site’s geographical location and a vector map outlining the coastal waters in BC (www.diva-gis.org/gdata). The seaway distances were stored in a matrix and later retrieved for further analyses.

These three data sets were merged into one table for statistical analyses. To achieve this, the wild data were used as the basis and farm data were combined with those using a unique identification for each time point (i.e. year-month). Therefore, each site-year-month of wild data was matched with up to five farm-year-month data points and thereafter with the relevant seaway distance data (for each site-farm pairing).

### Variables of interest

To evaluate the association between sea lice infestation levels on farmed and wild (Chum) salmon, it was hypothesised that the *L. salmonis* prevalence on out-migrating Chum in a certain month (t) and sampling site (i) was a function of the sum of the weighted (by seaway distances) load of adult female lice on each of the five farms (output pressure) one month prior; i.e. at (t-1). This 1-month lag time was applied in order to approximate the average time needed for the development from sea lice eggs produced by adult females on farmed fish to the attached stages on wild salmon (Equation 1). To build the final models, the following variables were defined and used:

- Y_i,t_ (the outcome of interest): the prevalence of infestation with lice (at any life stages) on the sampled out-migrating Chum salmon at time ‘t’ (per site-year-month of sampling), calculated as the number of Chum with at least one louse at time period ‘t’ in sampling site ‘i’ divided by the total Chum sampled at the same time period and site.
- L_j,t-1_: the abundance of adult female *L. salmonis* on farm ‘j’ at time ‘t-1’, calculated as the total number of adult female lice at time ‘t-1’ on farm ‘j’ divided by the number of fish sampled at the same time period and farm.
- N_j,t-1_: the average number of fish present on farm ‘j’ at time ‘t-1’.
- d_i,j_: seaway distance (km) between each pair of wild site ‘i’ and farm ‘j’.
- W_i,j_: Gaussian kernel density estimated weight for the seaway distance ‘d_i,j_’.
- Year: sampling year (for farmed and wild fish); 2007–2016 (n = 10).
- Month: sampling month; limited to March–June (n = 4).
- i: wild sampling site; W1–W16 (n = 16).
- j: farm; F1–F5 (n = 5).

To define our main predictor of interest (X _i,t_), the following formula was used:

1 $${X}_{i,t}={\sum }_{J=0}^{n}{\rm{W}}({d}_{i,j})\times {{\rm{L}}}_{j,t-1}\times {N}_{j,t-1}.$$

Where, X _i,t_ is the overall (at the inlet-level) lice pressure received by a wild site ‘i’ at time ‘t’ from the neighboring farm/s ‘j’; ‘n’ is the number of farms (n = 5) located within a radius (i.e. bandwidth) of 30 km from a wild sampling site. In the study region, all of the farms were located within the 30 km bandwidth from every wild site (Fig. 1); therefore, ‘n’ was consistently equal to five. The 30 km bandwidth was chosen based on both biological plausibility and statistical considerations. The expected traveling distance for sea lice particles from a source farm to its surrounding water environment has been investigated in previous studies^33,34^. With respect to statistical considerations, we examined a plausible range of bandwidths (5, 10, 20, 30, 40, 50, 60, and 100 km) to find the best fit model/s (results not shown). ‘L_t-1’_, ‘N_t-1’_, and d_i,j_ were defined earlier, under the variables of interest. W(d_i,j_) or the Gaussian kernel density weight for ‘d_i,j_’ was calculated using Equation 2:

2 $$W({d}_{i,j})=\frac{1}{\sqrt{2\pi }\,\sigma }\,{e}^{-\frac{di,{j}^{2}}{2{\sigma }^{2}}}$$

where, π = 3.1416; and σ is standard deviation or ¼ of bandwidth = 7.5 km.

For illustrative purposes only, the kernel densities shown in Fig. 1 were calculated by simulating a point process to represent a kernel density with a 30 km bandwidth. The points were simulated with ‘splancs’ package (www.maths.lancs.ac.uk/~rowlings/Splancs), and the kernel density surface with ‘spatstat’ package (www.spatstat.org) in R^50^, and the maps were generated with QGIS^51^.

### Descriptive statistics

For the farm-level data (n = 410), the annual abundance of adult female sea lice (i.e. the mean number of adult female *L. salmonis* per sampled fish) per farm during the study period were calculated (Table 1). The abundances of adult female lice at the inlet level for the period ‘t-1’ (February–May) in every year were calculated and graphed (n = 140; Fig. 2a).

The farms were active at various time periods between 2007 and 2016. The total number of active production cycles for the farms F1–F5, were 4, 5, 3, 3, and 5, respectively. Whenever a farm was inactive (fallowed), the output pressure from that farm (W(d_i,j_) × L_t-1_ × N_t-1_ = 0) did not contribute to the X_i,t_ calculations. If the lice count during the months of interest for a farm (February-May) was missing, the X_i,t_ including that farm was treated as missing (n = 85) and dropped from the final modeling process to prevent any potential biases.

For the wild salmon data (n = 365), the number of sampled fish and the number of fish with at least one sea louse attached during the study period were calculated (Table 2). The sampling was carried out in each wild site at various time points between 2007 and 2016 (Table 2). The monthly prevalences of sea lice infestation on Chum in the region during the out-migration season per year were calculated and graphed (Fig. 2b) to be compared with the corresponding farm abundances (Fig. 2a).

The frequency distribution of our main predictor of interest (X_i,t_) and the outcome (Y) for the final dataset (n = 280) were produced (Figs 3 and 4, respectively). All of the statistical analyses were carried out in Stata v15 (College Station, Texas, USA).

### Analytical statistics (modeling)

Due to excessive number of zeros (185 out of 280) in the *L. salmonis* prevalence on out-migrating Chum (Fig. 4), the effect of X_i,t_ on Y was evaluated using two different models to obtain maximum information from the data. First, a mixed-effects logistic regression model (model 1) was built, with Y being either zero (if prevalence = 0) or ‘one’ (if prevalence > 0) at each given wild site-year-month (n = 280). Second, for the non-zero prevalences (n = 95), a mixed-effects linear regression model (model 2) was built to further examine the association between X_i,t_ and Y, with Y being the prevalence of infestation (a continuous outcome), if present. The random effects of years and fixed effects of months were included in both models (random intercept models) to account for the potential confounding effects of time. Moreover, the interaction between X_i,t_ and month, as well as the effect of wild sampling sites on Y, were examined.

### Data availability

The datasets used during the current study are not publicly available due to confidentiality considerations, but can be provided by the corresponding author upon reasonable request.
